# Identification of a peptide antagonist of the FGF1–FGFR1 signaling axis by phage display selection

**DOI:** 10.1002/2211-5463.12618

**Published:** 2019-04-09

**Authors:** Magdalena Lipok, Anna Szlachcic, Kinga Kindela, Aleksandra Czyrek, Jacek Otlewski

**Affiliations:** ^1^ Department of Protein Engineering Faculty of Biotechnology University of Wroclaw Poland; ^2^ PORT - Polish Center for Technology Development Wroclaw Poland

**Keywords:** FGF1, FGFR1, fibroblast growth factor receptor 1, inhibitor, peptide, phage display

## Abstract

Overexpression of fibroblast growth factor receptor 1 (FGFR1) is a common aberration in lung and breast cancers and has necessitated the design of drugs targeting FGFR1‐dependent downstream signaling and FGFR1 ligand binding. To date, the major group of drugs being developed for treatment of FGFR1‐dependent cancers are small‐molecule tyrosine kinase inhibitors; however, the limited specificity of these drugs has led to increasing attempts to design molecules targeting the extracellular domain of FGFR1. Here, we used the phage display technique to select cyclic peptides F8 (ACSLNHTVNC) and G10 (ACSAKTTSAC) as binders of the fibroblast growth factor 1 (FGF1)–FGFR1 interface. ELISA and *in vitro* cell assays were performed to reveal that cyclic peptide F8 is more effective in preventing the FGF1–FGFR1 interaction, and also decreases FGF1‐induced proliferation of BA/F3 FGFR1c cells by over 40%. Such an effect was not observed for BA/F3 cells lacking FGFR1. Therefore, cyclic peptide F8 can act as a FGF1–FGFR1 interaction antagonist, and may be suitable for further development for potential use in therapies against FGFR1‐expressing cancer cells.

**Abbreviations**
Cys: cysteine
ERK1/2: extracellular signal‐regulated kinases 1/2
FDA: The Food and Drug Administration
FGF: fibroblast growth factor
FGFR: fibroblast growth factor receptor
Fmoc: fluorenylmethyloxycarbonyl protecting group
HRP: horseradish peroxidase
mAB: monoclonal antibody
PFU: plaque forming units
RT: room temperature
TMB: 3,3′,5,5′‐tetramethylbenzidine

Fibroblast growth factor receptor (FGFR) 1, a member of the fibroblast growth factor (FGF) receptor family, is one of the emerging targets for directed cancer therapies. FGFR1 is a receptor tyrosine kinase and as such consists of an extracellular domain that binding the ligands, a single transmembrane region and an intracellular tyrosine kinase domain 1. Ligands activating FGFR1 belong to FGF family and include FGF1, FGF2, FGF4, FGF9 and FGF21, with the majority of them involving heparin sulfate cofactor for interaction with FGFR1, causing receptor dimerization, transphosphorylation and signal transduction to the nucleus 2. In physiological conditions FGFR protein is responsible for regulation of many processes such as cell proliferation, migration, differentiation and survival 3. Due to their function, FGFRs can also influence tumor growth and stimulate angiogenesis, either by aberrant signaling or by overexpression on a cancer cell's surface 4. FGFR1 aberrations are observed in many cases of lung, head and breast cancer, as well as in neck squamous cell carcinoma or osteosarcoma 5.

Until now, no FGFR‐targeted cancer treatment has been approved for therapy; however, there are numerous small‐molecule tyrosine kinase inhibitors, anti‐FGF/FGFR monoclonal antibodies and FGF traps at various stages of preclinical and clinical development 6, 7, 8. Small‐molecule inhibitors, mainly directed to the intracellular tyrosine kinase domain, are currently the largest studied group. AZD4547, BGJ398 and JNJ‐42756493 are promising pan‐FGFR inhibitors and are now undergoing clinical trials on patients who suffer from different FGFR1‐dependent cancers 9, 10. A recently described small‐molecule inhibitor, SSR128129E, is a rare example of a small‐molecule drug binding to the extracellular domain of FGFR and allosterically preventing its activation 11, 12.

Antibodies and antibody fragments are also promising FGFR blockers, as in the case of FGFR1‐targeting scFvD2‐Fc , which both alone and coupled with the cytotoxic drug monomethyl auristatin E, showed very strong inhibition of FGFR1‐dependent lung cancer cell lines 13. Cytotoxic drug conjugation was also used in designing FGF1 or FGF2–cytotoxic drug conjugates. Coupling toxic chemical compounds with natural FGFR1 ligands allowed specific binding to the receptor and direct internalization into cancer cells overexpressing FGFR1 or FGFR2 14, 15.

In this rapidly developing landscape of FGFR‐targeted therapeutic approaches there are only a few reports on the use of peptides with FGFR1 binding properties. A short peptide (VYMSPF) has been identified by phage display screening against cells expressing FGFR1 that showed high FGFR1 inhibition ratio and was able to block receptor activation in a dose‐dependent manner 16. Similarly, a MQLPLAT peptide found by phage display showed accumulation in FGFR‐expressing gastric carcinomas, and was described as a promising targeting agent, i.e. for cancer‐selective gene therapies; however, it was not specific to one of the four FGFR forms (FGFR1–4) 17.

Due to their small size, peptides are known to be rapidly digested by proteolytic enzymes and disposed of in the bloodstream. However, thanks to fast and cheap synthesis they are still highly important players in drug discovery 18. Over many years researchers have developed various methods that allow for fast selection and modification of peptides in an attempt to improve their biological activity. Small changes in peptide structure such as incorporation of unnatural amino acid, cyclization or peptide‐protein conjugation are often used for potential therapeutic peptides 19. One of the features of a peptide that can have a dramatic effect on both peptide stability and affinity to target is cyclic form. In comparison with the linear form, the rigid conformation of cyclic peptides is beneficial, causing decreased change in the entropy of target binding. The cyclic peptides are also favored due to their similarity to naturally occurring protein ligands 20. Moreover, many protein binders adopt secondary or tertiary structure, with peptide loops playing often a pivotal role in protein–protein interaction. Therefore, designing cyclic peptides can bring promising results in the peptide–protein binding search 21.

Since cyclization of a target‐binding peptide can lead to loss of its affinity to target, rather than cyclizing binding peptides we have chosen to screen a library of cyclic peptides, with N and C termini linked by a Cys–Cys disulfide bond. Here, we present a FGFR1‐binding cyclic peptide showing the ability to suppress FGFR1‐induced cell proliferation.

## Materials and methods

### Materials

The Ph.D‐C7C Phage Display Peptide Library Kit (no. E8120S) was purchased from New England Biolabs (NEB; Ipswich, MA, USA). The fully glycosylated extracellular domain of FGFR1 IIIc fused to the Fc domain of human IgG1 (FGFR1‐Fc) was produced as described previously 22. The Fc fragment was cloned, expressed, and purified in the same manner as FGFR1‐Fc. Recombinant FGF1 (Met‐Ala‐FGF1^22–155)^ was produced in an *Escherichia coli* BL21(DE3)pLysS strain at 37 °C and purified on a heparin–Sepharose CL‐6B resin according to described protocols 23, 24.

### Phage display *in vitro* biopanning

A 96‐well plate was coated with 100 μg·mL^−1^ FGFR1‐Fc overnight at 4 °C in 0.1 m NaHCO_3_ (pH 8.6) and then blocked with BSA for 2 h at 4 °C. Afterwards the wells were washed five times with TBST (0.1% Tween‐20 in Tris‐buffered saline). The original library was diluted in TBST (final concentration 2 × 10^11^ plaque forming units, PFU) and 100 μL was added to each coated well for 2 h at 4 °C with gentle agitation. After washing the plate 10 times with TBST, the bounded phages were eluted with 0.2 m glycine/HCl (pH 2.2) and neutralized with 1 m Tris/HCl (pH 9.1). The eluted phages were then amplified, precipitated with polyethylene glycol/NaCl and titrated according to standard protocol (NEB). In the second round of selection there was an additional counterselection step for the Fc fragment. Fc at a concentration of 50 μg·mL^−1^ was immobilized in wells and blocked with 5% non‐fat milk. Subsequently, the phages (2 × 10^11^ PFU) were incubated with Fc for 1 h at 4 °C. Afterwards, phage clones were transferred to wells with FGFR1‐Fc (70 μg·mL^−1^) and incubated for 1 h at 4 °C with gentle agitation. After each round of selection, amplified phages were diluted and used for the next round of biopanning. In the third round of selection, the concentration of immobilized FGFR1‐Fc and Fc was decreased down to 50 and 20 μg·mL^−1^, respectively. Additionally after the last round of selection, binding phages were eluted with 100 molar excess of FGF1 over applied phage library (2 × 10^11^ PFU).

### ELISA screening

ELISA was conducted after the third round of selection. The 96‐well Maxisorp F plate was coated with FGFR1‐Fc (5 μg per well), incubated at 4 °C overnight and additionally blocked with 3% BSA for 2 h at 4 °C. After washing with TBST (0.2% Tween‐20) inoculated phage clones were added to the wells and incubated for 2 h at 4 °C. Afterward the plate was washed five times and horseradish peroxidase (HRP)–anti‐M13‐monoclonal antibody (mAB) (1 : 5000 v/v, no. 27‐9421‐01; GE Healthcare, Chicago, IL, USA) was added to each well and incubated at room temperature (RT) for 1 h. Subsequently, the plate was washed four times and 3,3′,5,5′‐tetramethylbenzidine (TMB) was used for detection with the absorbance measured at 450 nm. Similarly, for the estimation of the level of Fc‐binding, the plate was coated with Fc (5 μg per well) and treated with phage clones as above.

### Quantitative ELISA

The 96‐well plate was coated with 50 μg·mL^−1^ FGFR1‐Fc overnight at 4 °C in 0.1 m NaHCO_3_ (pH 8.6), washed three times with PBS and subsequently blocked with 3% BSA for 2 h at 4 °C. Subsequently, the wells were washed five times with PBS and 100 μL of F8 and G10 phage clones (10^9^ PFU) was added for 1 h at RT with gentle agitation. Afterwards the plate was washed five times with PBS and the HRP–anti‐M13‐mAB (1 : 5000 v/v) was added and incubated at RT for 1 h. Next, the plate was washed 10 times with PBS and substrate TMB was used for detection with the absorbance measured at 450 nm. Measurements were performed three times, each time in triplicate.

### Competitive ELISA

An ELISA plate was coated with FGFR1‐Fc as for quantitative ELISA. The wells were washed five times with PBS and 100 μL of FGF1 (10 μg·mL^−1^) and phage clone (10^9^ PFU) mix or phage alone was added for 1 h at 4 °C with gentle agitation. After incubation the plate was washed five times with PBS and the detection with HRP–anti‐M13‐mAB (1 : 5000 v/v) and TMB substrate was performed as before. Measurements were performed three times, each time in triplicate.

### Peptide synthesis and disulfide bridge formation

Peptides were synthesized manually with C‐terminal amidation on Fmoc‐solid phase according to standard strategy. The purity of obtained products was verified using RP‐HPLC and the proper molar masses of synthesized peptides were confirmed by mass spectrometry.

Disulfide bridge formation was optimized and oxidation folding in redox buffer protocol proved to be the most efficient method. Peptides at a final concentration of 0.1 mg·mL^−1^ were dissolved in Milli‐Q water (Merck Millipore; Burlington, MA, USA) at pH 3 and mixed with 4 m urea, 300 μm reduced glutathione and 150 μm oxidized glutathione. The pH was then adjusted to 8.7 with 1 m Tris/HCl and the solution was left overnight at RT with gentle stirring. Finally, cyclic peptides were purified via RP‐HPLC and intramolecular disulfide bond formation was confirmed with matrix‐assisted laser desorption/ionization time‐of‐flight mass spectrometry.

### Cell culture

Mouse embryo fibroblast cells, NIH 3T3 (ATCC no. CRL‐1658), were obtained from American Type Culture Collection (ATCC, Manassas, VA, USA). NIH 3T3 cells were cultivated in Dulbecco's modified Eagle's medium without sodium pyruvate (Thermo Fisher Scientific, Waltham, MA, USA) supplemented with 10% fetal bovine serum (Thermo Fisher Scientific) and antibiotics (100 U·mL^−1^ penicillin, 100 μg·mL^−1^ streptomycin). A murine pro B cell line, BA/F3, was purchased from Leibniz Institute DSMZ‐German Collection of Microorganisms and Cell Cultures (DSMZ, Braunschweig, Germany), and a BA/F3 FGFR1c cell line stably transfected with the FGFR1 gene (isoform IIIc) was provided by D. M. Ornitz (Washington University, St Louis, MO, USA). Both, BA/F3 and BA/F3 FGFR1c cells were cultivated in RPMI‐1640 medium (Thermo Fisher Scientific) supplemented with 10% newborn bovine calf serum (Thermo Fisher Scientific), antibiotics (100 U·mL^−1^ penicillin, 100 μg·mL^−1^ streptomycin), β‐mercaptoethanol (50 nm) and mouse interleukin 3 (PeproTech, New Jersey, NJ, USA).

### Western blot analysis of inhibition of FGF1–FGFR1‐dependent signaling pathway

NIH 3T3 (1.5 × 10^5^ cell per well) cells were seeded in six‐well plates and then serum‐starved for 6 h. Cells were then treated with 40 μm peptides for 30 min prior to stimulation with 2 ng·mL^−1^ FGF1. After a 15 min incubation with FGF1, cells were lysed in Laemmli sample buffer. Cell lysates were subjected to SDS/PAGE separation and transferred to polyvinylidene difluoride membrane, which was probed with primary antibodies [anti‐phospho‐extracellular signal‐regulated kinases 1/2 (ERK1/2) rabbit mAB (no. 9101; Cell Signaling Technology, Leiden, The Netherlands), anti‐phospho‐FGFR1 mouse mAB (no. 3476; Cell Signaling Technology), anti‐ERK1/2 mouse mAB (no. 05‐1152; Millipore, Burlington, MA, USA), anti‐FGFR1 rabbit mAB (no. 9740; Cell Signaling Technology) and anti‐tubulin mouse mAB (no. T6557; Sigma‐Aldrich, Saint Louis, MO, USA)] overnight at 4 °C, followed by donkey anti‐rabbit (no. sc‐2313; Santa Cruz Biotechnology, Dallas, TX, USA) or donkey anti‐mouse IgG (no. sc‐2318; Santa Cruz Biotechnology), HRP‐conjugated antibody (1 : 5000 v/v) and detection with a chemiluminescent substrate.

### Inhibition of cell proliferation assay

BA/F3 FGFR1c and BA/F3 cells were seeded in a 96‐well plate at a density of 3 × 10^4^ per well in RPMI‐1640 medium without interleukin 3. After 24 h, cells were treated with 2 ng·mL^−1^ FGF1 alone or with a series of concentrations of cyclic or linear peptide F8. After 48 h, cell viability was determined by addition of 10% (v/v per well) PrestoBlue Cell Viability Reagent (Invitrogen, Carlsbad, CA, USA) for 2 h. The fluorescence was measured at 560 nm using Infinite M1000 PRO, TECAN plate reader (Tecan Group Ltd. Männedorf, Switzerland). Similarly, to establish possible unspecific peptide toxicity towards cells, cells seeded in RPMI‐1649 medium supplemented with serum were treated with cyclic or linear F8 peptide and their viability was assessed as described above.

## Results

### Phage display *in vitro* biopanning

**Figure 1:**
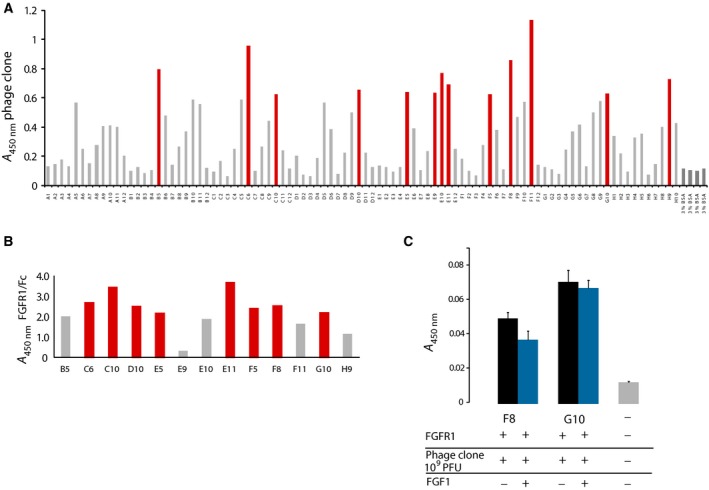
Screening and identification of FGFR1‐binding peptides. (A) ELISA screening of phages binding to FGFR1‐Fc eluted after third round of selection. Clones giving the highest signal (marked in red) were selected for further analysis. (B) Quantitative ELISA was performed with either immobilized FGFR1‐Fc or Fc, to determine peptide specificity towards FGFR1 (C) Competitive ELISA detected decreased F8 clone binding to FGFR1 in the presence of FGF1, suggesting the possibility of overlapping binding interface. The effect was not observed for G10 clone. Presented data are the mean absorbance values of triplicate measurements, and error bars correspond to SD.

In order to identify new peptide binders specific towards FGFR1, the Ph.D‐C7C Phage Display peptide library (NEB) was used. The library consists of 10^9^ randomized cyclic peptides, containing intramolecular disulfide bond linking N‐ and C‐terminal cysteines. For the selection of high‐affinity phage clones, the conditions of biopanning were differing between consecutive rounds of selection, to provide increasing stringency of FGFR1 selection (Table 1). The amount of immobilized target (FGFR1‐Fc) gradually decreased at each round of selection. Moreover, to prevent selection of clones binding the Fc region, and not FGFR1, after the second and third rounds of biopanning, additional counterselection with Fc fragment was performed. To maximize the chance that selected peptides bind on the ligand–receptor interface, in the last step of selection, FGFR1 binding phage clones were eluted with 100 molar excess of FGF1. The enrichment of FGFR1 binding phages for each panning round is presented in Table 1. Ninety‐two individual phage clones were tested in an ELISA (Fig. 1A). Thirteen clones presented a high target/negative control absorbance ratio and were considered as FGFR1‐binding positive. These positive phage clones were verified in ELISA with regard to the level of their binding of Fc fragment (Fig. 1B). Eight out of 13 tested phage clones showed significantly higher binding towards FGFR1 compared to Fc, suggesting specific binding to the FGFR1 portion of the target protein, and were sent for sequencing.

**Table 1:** Enrichment of FGFR1 binding phages for each panning round

Round of selection	FGFR1 (μg·mL^−1^)	Concentration of Tween‐20 (v/v)	Elution	Input phage (PFU)	Output phage (PFU)	Recovery (%)
1	100	0.1	0.2 m glycine pH 2.2	2 × 10^11^	1 × 10^7^	5 × 10^−3^
2	70	0.3	0.2 m glycine pH 2.2	2 × 10^11^	12 × 10^7^	6 × 10^−2^
3	50	0.5	0.2 m glycine pH 2.2/FGF1	2 × 10^11^	14 × 10^5^/8 × 10^4^	7 × 10^−4^/4 × 10^−5^

The results of DNA sequencing revealed that four of sequenced phage clones contained inserts encoding peptide sequence. The peptide sequences were respectively CSLNHTVNC for clone F8, CSAKTTSAC for clone G10 and CNAGHLSQC for clones D10 and E11 (Fig. 1C). Even though the CNAGHLSQC sequence occurred twice, it was not chosen for further analysis, as our experiments with Ph.D‐C7C library‐based selections against other targets indicated that this sequence is not a specific FGFR1 binder and is identified in multiple screens. It has also been reported in literature as a clone that is isolated from phage libraries but does not prove to be binding to the target; therefore, it may be a so‐called parasitic sequence that amplifies quickly and has a selective advantage 25, 26, 27. Ultimately, peptide sequences from clones F8 and G10 were chosen for further characterization.

### Competitive ELISA for selected phages

For a FGFR1‐binding peptide to have a therapeutic potential on its own, it has to interfere with binding of the ligand to the receptor and therefore inhibit FGF‐induced reaction of cells. Otherwise the binder can be used as a therapeutic targeting agent only if a cytotoxic or diagnostic payload is attached to it. In the last round of selection performed, phages were additionally eluted with excess of FGF1 protein. Due to the addition of that step, the phage clones that bind to the FGFR1 site responsible for interacting with FGF1 should be released.

In order to verify if clones F8 or G10 share similar binding site on FGFR1 as its ligand, FGF1, a competitive binding assay has been performed. To ensure the same concentration of interacting phages, clones F8 and G10 were amplified, purified and concentrated, and their PFU was established spectrophotometrically. A competitive binding assay in the presence or absence of FGF1 revealed that for clone F8 there is a minor, albeit statistically significant, decrease in peptide‐presenting phage binding upon addition of FGF1 (Fig. 1D). This effect was not observed in the case of clone G10. Such a result can be explained either by non‐overlapping binding sites for G10 peptide and FGF1, or that peptide G10 binds to FGFR1 with affinity so high that it completely precludes FGF1 binding. However, the latter explanation is much less probable in the case of peptidic binders, as their binding constants usually lie in the micromolar range. Ultimately, both peptides were chosen for synthesis and evaluation with regard to their ability to interfere with the FGF1‐induced cellular response.

### Peptide synthesis and cyclization

**Figure 2:**
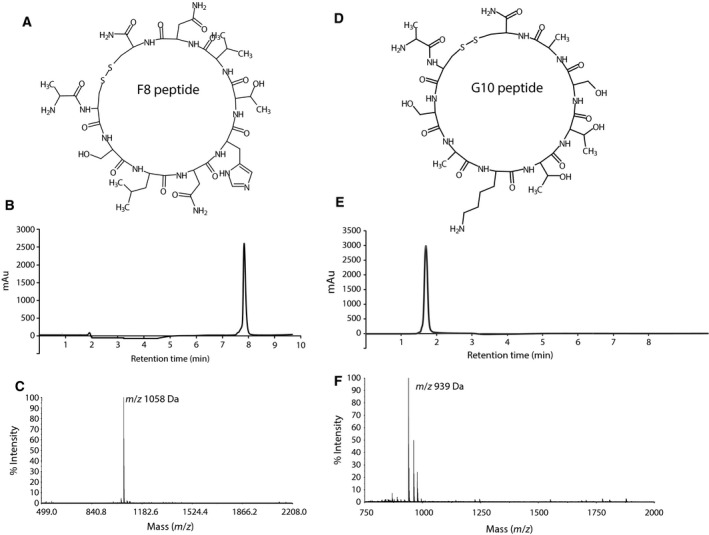
Chemical synthesis and cyclization of F8 and G10 peptides. Depicted is the chemical structure of F8 (A) and G10 (D) cyclic peptide. Chromatography profiles of purified peptides (B, E) show their chemical purity, and matrix‐assisted laser desorption/ionization mass spectrometry analysis confirms the disulfide bond formation and monomeric form (C, F).

F8 and G10 peptides were synthesized on solid‐phase resin according to standard F‐moc strategy and their purity and proper mass were confirmed by RP‐HPLC and electrospray ionization mass spectrometry measurements (F8, 1060 Da; G10, 941 Da). One of the main structural features of these peptides is their cyclic form; therefore, the efficient reduction of thiol groups in two terminal cysteine residues yielding the formation of a disulfide bridge is crucial for them to achieve their active conformation (Fig. 2A,D). We have tested three disulfide bond formation strategies: direct air oxidation, oxidation by DMSO and oxidation by reduced and oxidized glutathione 28, 29, 30. We found that direct air oxidation yields a significant amount of disulfide‐bridged dimeric and trimeric peptides, and performing the oxidation reaction at lower peptide concentrations did not decrease their ratio to monomeric peptide. In the case of DMSO‐mediated oxidation we encountered problems with chemical modifications of peptides, detected by molecular mass change.

Oxidation performed with the mixture of reduced and oxidized glutathione yielded best results, with little dimeric and trimeric species, which were removed during RP‐HPLC separation (Fig. 2B,E). Mass spectrometry measurements confirmed disulfide bond formation and the monomeric form of both cyclic peptides (Fig. 2C,F). To remove any traces of organic solvents, peptides were washed with buffer and lyophilized again for the use in cell culture experiments.

### F8 peptide inhibits FGFR1 downstream signaling

**Figure 3:**
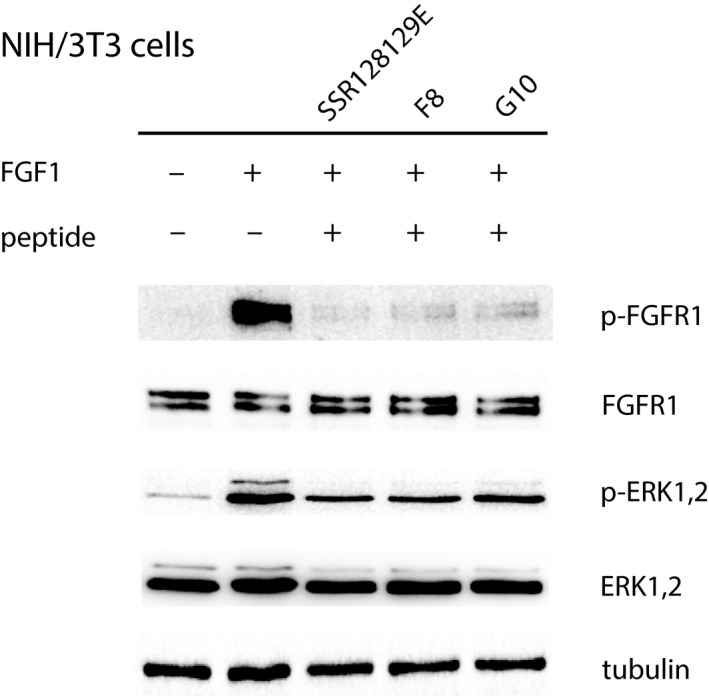
F8 and G10 peptides can block FGF1‐induced signaling in fibroblast cells. Mouse fibroblast NIH/3T3 cells were stimulated with FGF1 in the presence or absence of F8 peptide, G10 peptide or SSR128129E, an allosteric FGFR1 inhibitor. Protein lysates were subjected to immunoblotting analysis to detect activation of FGFR downstream signaling.

One of the highly sensitive readouts of FGFR1 binding to its ligand (here FGF1) is the resulting stimulation of receptor phosphorylation and subsequent activation of downstream signaling kinases. Their phosphorylation upon addition of FGF1 with or without the pre‐treatment of cells with F8 and G10 peptide was detected by western blot. We have used NIH 3T3 mouse fibroblast (Fig. 3), for which we observed activation of FGFR1 with FGF1 as detected with increased levels of phospho‐FGFR1 and phospho‐ERK1/2 kinase, with unchanged total levels of these proteins. FGF1 stimulation was inhibited by the addition of F8 and G10 peptide, as detected with the significant decrease in the amount of phosphorylated FGFR1. The effect was less pronounced when comparing the levels of downstream activation – (phospho‐ERK1/2 kinase), but still comparable with the effect of SSR128129E small molecule inhibitor described before 11, 12.

### F8 peptide inhibits FGF1‐induced cell proliferation

Obtained results suggest that F8 and G10 peptides can compete with FGF1 for binding to FGFR1. To evaluate if we can observe inhibition of the FGF1 long‐term cell response in the presence of these peptides, we have performed cell proliferation assays. As NIH 3T3 cells express all four FGF receptors (FGFR1–4), all of which are activated by FGF1, peptide‐caused inhibition of FGFR1 will not be reflected in inhibition of FGF1‐induced proliferation. Therefore, we have chosen to use cells expressing only FGFR1. We have used a set of BAF/3 and BAF/3 FGFR1c cells, with the latter stably transformed with FGFR1.

**Figure 4:**
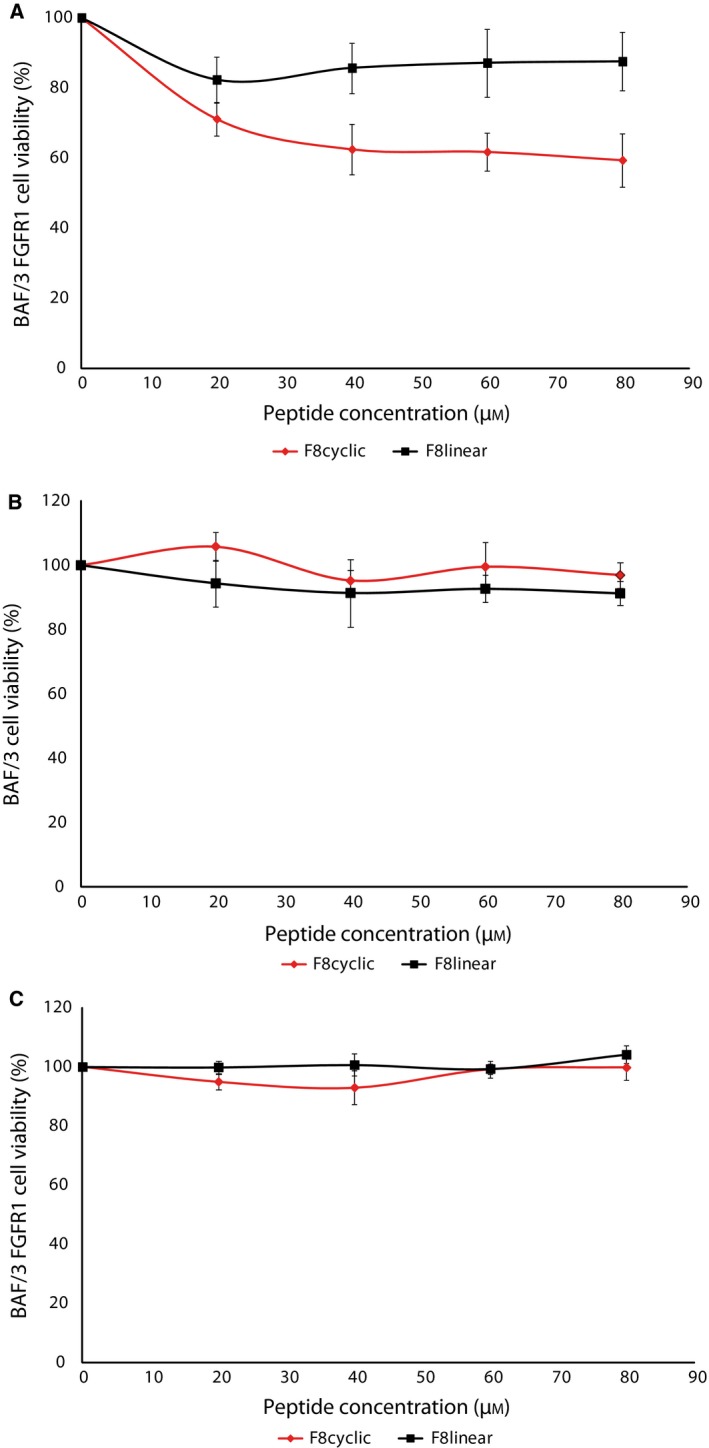
Antiproliferative activity of F8 peptide is FGFR1 dependent. (A, B) Concentration‐dependent inhibition of FGF1‐induced proliferation of BAF/3 FGFR1 cells (A) and BAF/3 cells (B) treated with cyclic and linear form of peptide F8. (C) F8 peptide, in either linear or cyclic form, does not show any cytotoxic effects on cells within tested concentration range. Data presented are the mean absorbance values of triplicate samples, and the error bars show standard deviation.

We tested both F8 and G10 peptides and observed that the latter did not show any inhibition of FGF1‐induced cell proliferation. Therefore, we focused on F8 peptide. A range of concentrations of F8 peptide was used together with FGF1. As a control, we have used a linear F8 peptide, which has the same amino acid sequence but without its cyclic conformation forced by disulfide bridge. The results demonstrated that cyclic peptide F8 possesses the ability to decrease cell proliferation by over 40% in relation to cells treated only with FGF1 (Fig. 4A). The linear form of peptide F8 did not show any inhibitory effect of cell proliferation, which is an indication that only the cyclic form is able to interact with FGFR1. The inhibitory effect was specific to cells expressing FGFR1, as the peptides, both cyclic and linear, had no effect on FGF1‐induced proliferation of BAF/3 cells, lacking any FGF receptors on their surface (Fig. 4B). Moreover, to confirm that the inhibitory effect of peptide F8 was not due to the unspecific cell toxicity, we treated BAF/3 FGFR1 cells with both linear and cyclic F8 peptide in the absence of FGF1, and did not observe any decrease in viability, indicating lack of unspecific chemical cytotoxicity (Fig. 4C).

## Discussion

Peptide drugs and their modified counterparts are increasingly approved by the Food and Drug Administration (FDA). Between 2000 and 2016, 28 new peptide drugs have appeared on the worldwide pharmaceutical market. At the moment over 150 new peptide drug candidates are being tested in clinical trials, and many more are being advanced 31.

Screening/selection from phage display libraries is widely used for identification of new peptides for pharmacological applications. For example, Romiplostim (Nplate) was developed by Amgen Inc. 32. The peptide identified by phage display screening and after reformatting into Fc‐fusion protein (peptibody), was approved in 2008 by the FDA for treatment of chronic idiopathic thrombocytopenic purpura.

As a molecular target, we have chosen FGFR1, since it can regulate tumor growth and stimulate angiogenesis, either by aberrant signaling or by overexpression on the cancer cell surface, as reported for multiple lung and breast cancers. To date, a few peptides targeting FGFRs or FGFs have been reported. In one of the first approaches, Kavanaugh and colleagues selected a 26‐amino‐acid peptide via phage display screening and further improved its binding properties by fusing it with a dimerization domain. Such a dimer showed very high affinity and was able to reproduce FGF activity, acting as a functional agonist 33. In 2002, Roeske and colleagues identified a 6‐amino‐acid linear peptide targeting FGFR1. Peptide VYMSPF was predicted by computational docking to bind to the hydrophobic surface of FGFR1. Moreover, a cell proliferation assay on an NIH 3T3 cell line indicated that VYMSPF shows an ability to inhibit mitogenic activity of FGF1 in a dose‐dependent manner 16. Similarly, in a study where a 12‐amino‐acid peptide library was screened against FGFR3, VSPPLTLGQLLS peptide was identified as binding specifically to the extracellular domain of this receptor 34.

A thematic approach to dissociate the FGF–FGFR interaction relies on selection of peptides that are able to bind to FGF and block its interaction with FGF receptor. A 15‐amino‐acid FGF1‐binding peptide was discovered that had an ability to inhibit mitogenic activity of FGF1 35. Another peptide (APDTKTQ) was identified by phage display from a library of 7‐amino‐acid linear peptides and was evaluated in tests with breast cancer cell lines overexpressing FGFR. The data indicate that the peptide was able to inhibit cell proliferation and cell cycle progression 36.

In the present study, we used the C7C Phage Display peptide library for selection of an FGFR1‐binding peptide that act as an antagonist of the FGF1–FGFR1 interaction. We chose to select from a library of cyclic peptides, to use their advantageous stability and decreased entropic effects favouring stronger binding to target. As a result, we selected three peptide sequences. One of the selected clones (A3) turned out to be described previously as a parasitic clone that amplifies preferentially without affinity to protein target, and therefore was not chosen for further research. ELISA data obtained for two other clones showed that the F8 (ACSLNHTVNC) clone is characterized by higher inhibition ratio of FGF1–FGFR1 binding than peptide G10 (ACSAKTTSAC) (Fig. 1D). Sequence similarity searches did not reveal any direct resemblance of selected peptides to either FGF family ligands or FGFR receptors. This is not entirely surprising as the circular conformation of the peptide cannot be directly related to the linear protein sequence.

We synthesized both F8 and G10 peptides and cyclized them via intramolecular disulfide bond formation. To obtain the monomeric form of cyclic peptides, we decided to test three different cyclization protocols. Ultimately, we applied oxidation performed with the mixture of reduced and oxidized glutathione, which yielded the highest amount of the monomeric form of cyclic peptides.

We tested if F8 and G10 possess the ability to inhibit FGFR downstream signaling and FGF1‐induced cell proliferation. F8 peptide inhibited downstream signal transduction in a dose‐dependent manner much more strongly than G10.

The cyclic F8 peptide had the ability to decrease cell proliferation by over 40% in relation to a positive control (FGF1 alone). Importantly, a linear form of F8 did not possess the ability to inhibit the FGF1–FGFR1 interaction, indicating that only a cyclic form of F8 peptide can block ligand–receptor binding. This effect was specific to cells expressing FGFR1 (BA/F3 FGFR1c), since untransfected BA/F3 cells lacking FGF receptors did not show any inhibitory effects. F8 did not show any cell toxicity effect on tested cell lines, which makes it a potential peptide drug candidate. Thus, peptide F8 in its cyclic form can act as an FGF1–FGFR1 inhibitor, which can be used in a further survey for use in medical application.

## Conflict of interest

The authors declare no conflict of interest.

## Author contributions

JO designed and supervised the project; ML and AS designed the experiments, prepared the figures, and wrote the manuscript; ML, KK and AC performed the experiments; and ML, AS and JO analyzed the data, discussed results from the experiments, and edited the manuscript.
